# Prognostic value of albumin-related inflammatory ratios for 30-day mortality in rheumatic heart disease: a dual-cohort study with external validation

**DOI:** 10.3389/fnut.2026.1916028

**Published:** 2026-08-13

**Authors:** Wen Li, Zhanfang Zhu, Jinxia Hao, Yunhui Zheng, Ruochen Zhang, Xiaoxiang Liu, Ying Lv, Fuqiang Liu, Na Zhu

**Affiliations:** 1 Department of Clinical Trials, Shaanxi Provincial People’s Hospital, Xi’an, Shaanxi, China; 2 Department of Internal Medicine, Xi’an Jiaotong University Hospital, Xi’an, Shaanxi, China; 3 Department of Cardiology, Shaanxi Provincial People’s Hospital, Xi’an, Shaanxi, China

**Keywords:** albumin-related inflammatory biomarkers, external validation, red cell distribution width-to-albumin ratio, rheumatic heart disease, short-term mortality

## Abstract

**Background:**

Rheumatic heart disease (RHD) is a chronic valvular condition characterized by persistent autoimmune inflammation and nutritional deficiencies. Albumin not only reflects the body’s nutritional status but also eliminates pro-inflammatory stimuli and mitigates inflammatory responses. This study aimed to evaluate the prognostic value of novel albumin-related inflammatory biomarkers for short-term mortality in patients with RHD.

**Methods:**

This retrospective dual-cohort study included 496 RHD patients from the MIMIC-IV database and 268 patients from Shaanxi Provincial People’s Hospital. The primary outcome was all-cause mortality within 30 days. Cox proportional hazards regression, restricted cubic spline analysis, time-dependent ROC curves, and integrated discrimination improvement (IDI) were performed to assess the independent associations, dose–response relationships, discriminative ability, and incremental predictive value of each biomarker.

**Results:**

RAR consistently demonstrated the strongest association with 30-day mortality across both cohorts. In fully adjusted Cox models, RAR remained significantly associated with mortality (MIMIC: HR 4.12, 95% CI 1.79–9.49; external validation: HR 3.20, 95% CI 1.25–8.23). RAR also showed significant positive linear dose–response relationships, achieved the highest time-dependent AUC, and provided significant incremental predictive value beyond SOFA, SAPS II, and OASIS in both cohorts (all IDI *p* < 0.05). LAR was protective in the validation cohort (HR 0.61, 95% CI 0.43–0.88), while NAR and PAR showed cohort-specific associations. MAR demonstrated limited prognostic value.

**Conclusion:**

RAR has been confirmed as the most reliable indicator for predicting 30-day mortality in RHD patients. It offers a simple, cost-effective tool for early risk stratification, particularly in resource-limited settings where RHD burden remains highest.

## Introduction

Rheumatic heart disease (RHD) is a chronic valvular heart disease caused by acute rheumatic fever triggered by Group A streptococcal infection. It is one of the leading causes of morbidity and mortality among adolescents and young adults worldwide, particularly in low- and middle-income countries (1, 2). Although its incidence has significantly declined in developed countries, RHD remains a substantial public health burden in resource-limited regions, contributing to high cardiovascular disease incidence and mortality (3, 4). In developed countries where acute rheumatic fever has been largely eradicated and in healthcare systems undergoing transition, RHD primarily affects elderly patients who contracted the disease during childhood; these patients often develop late complications in their later years (5, 6). Although treatments such as cardiac surgery can improve outcomes in patients with severe RHD, overall mortality rates remain high (7, 8). Current clinical risk assessments primarily rely on clinical symptoms, echocardiographic parameters, and complications (9). The identification of novel, readily available prognostic biomarkers that comprehensively reflect the underlying pathogenic mechanisms remains a critical unmet clinical need.

Malnutrition is prevalent among RHD patients and has been established as an independent risk factor for mortality in pediatric RHD patients (10). Serum albumin not only reflects the body’s nutritional status but also eliminates pro-inflammatory stimuli and mitigates inflammatory responses (11, 12). Therefore, combining inflammatory markers with albumin levels may provide a more comprehensive assessment of the “inflammatory-nutritional imbalance” state in RHD patients. Previous studies have demonstrated the predictive value of albumin-related inflammatory markers in diseases such as lung cancer, breast cancer, and HELLP syndrome (13–15). In the field of cardiovascular diseases, albumin-related inflammatory markers have also been shown to correlate with conditions including coronary heart disease, diabetes mellitus, heart failure, myocardial infarction, and cardiac arrest (16–20). However, there is currently a lack of comprehensive, systematic research evaluating the prognostic value of these novel albumin-related inflammatory ratios (NAR, LAR, MAR, PAR, and RAR) for mortality in the RHD population.

Therefore, this study analyzed data from the Medical Information Mart for Intensive Care-IV (MIMIC-IV) database and an independent external validation cohort from a Chinese hospital to investigate the associations of NAR, LAR, MAR, PAR, and RAR with mortality in patients with RHD. We aimed to identify the most predictive indicator and thereby provide a simple, cost-effective tool for risk stratification in this population.

## Method

### Data sources

The data for this study were obtained from the publicly available MIMIC-IV (21). MIMIC-IV is a large, single-center, publicly accessible clinical database for intensive care units, containing anonymized clinical data from hospitalized patients at Beth Israel Deaconess Medical Center in the United States between 2008 and 2019. The MIT Institutional Review Board approved the establishment of this database, and all patient data were de-identified. Therefore, no additional ethical review approval was required for this retrospective data analysis. For external validation, an independent retrospective cohort was obtained from the Shaanxi Provincial People’s Hospital, comprising consecutive RHD patients admitted to the ICU between June 2020 and June 2025. Clinical data were extracted from the hospital electronic medical record system. As this study was a retrospective investigation with all data anonymized, our hospital waived ethical review requirements and did not require informed consent forms to be signed. This study was conducted in accordance with the Declaration of Helsinki.

### Study population

**Figure 1:**
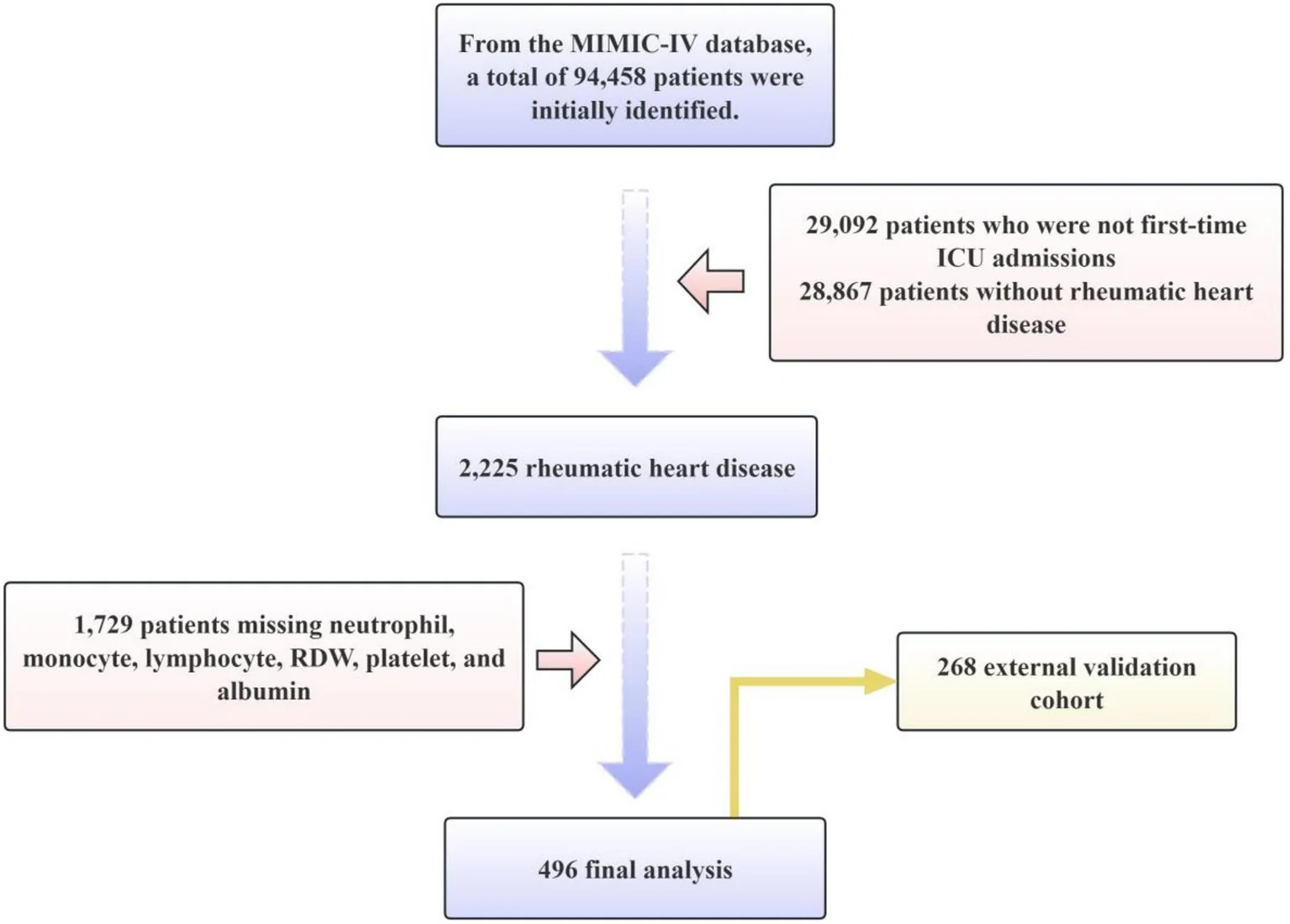
The flowchart of patient selection.

This retrospective cohort study employed dual-cohort design. For the MIMIC-IV cohort, adult patients diagnosed with RHD at their first ICU admission were initially identified (*n* = 2,225). Rheumatic heart disease was defined by ICD-9 codes 393–398 or ICD-10 codes I05-I09. After excluding 1,729 patients with missing data for key variables (albumin, neutrophil count, lymphocyte count, monocyte count, platelet count, and red blood cell distribution width), 496 patients were ultimately included (Figure 1). For the external validation cohort, 268 consecutive RHD patients admitted to the ICU at Shaanxi Provincial People’s Hospital between June 2020 and June 2025 were enrolled.

### Covariates

To control potential confounders, we collected the following covariates in both cohorts. In the MIMIC cohort, demographic characteristics, laboratory parameters, vital signs, disease severity, comorbidities, and therapeutic interventions were collected. Demographic characteristics included age, gender, and weight. Laboratory parameters comprised serum sodium, potassium, calcium, anion gap, blood urea nitrogen, glucose, creatinine, albumin, neutrophil count, lymphocyte count, monocyte count, platelet count, and RDW. Vital signs, including respiratory rate, systolic blood pressure (SBP), diastolic blood pressure (DBP), and heart rate. Disease severity was assessed using the Sequential Organ Failure Assessment (SOFA), Simplified Acute Physiology Score II (SAPS II), and Oxford Acute Severity of Illness Score (OASIS). Comorbidities were defined according to ICD codes and included hypertension, diabetes, malignant tumor, kidney disease, sepsis, chronic pulmonary disease, and congestive heart failure. Therapeutic interventions during hospitalization, including antibiotic use and valve surgery (yes/no). In the validation cohort, the same set of covariates was collected where applicable. For both cohorts, laboratory parameters were derived from the initial test results obtained within 24 h after patient admission to the ICU. This approach was selected to accurately reflect the pathophysiological state of patients when critical symptoms emerged and to minimize the influence of treatment-related factors on laboratory parameters.

No missing values were observed for the included variables in the external validation cohort. Variables with a missing proportion exceeding 20% were excluded from the analysis in MIMIC cohort, multiple imputation was performed using the random forest algorithm for covariates with less than 20% missing values, the specific missing proportions for each covariate in MIMIC cohort are provided in Supplementary Table S1.

### Exposure and outcome

The exposure variables comprised five albumin-related inflammatory ratios, calculated as follows: neutrophil-to-albumin ratio (NAR) = log[neutrophil count (×10^9^/L) / albumin (g/dL)]; lymphocyte-to-albumin ratio (LAR) = log[lymphocyte count (×10^9^/L) / albumin (g/dL)]; monocyte-to-albumin ratio (MAR) = log[monocyte count (×10^9^/L) / albumin (g/dL)]; platelet-to-albumin ratio (PAR) = log[platelet count (×10^9^/L) / albumin (g/dL)]; and red blood cell distribution width-to-albumin ratio (RAR) = log[red blood cell distribution width (%) / albumin (g/dL)]. For both cohorts, laboratory parameters were obtained from the first measurements within 24 h of ICU admission. The primary outcome was all-cause mortality within 30 days after ICU admission.

Logarithmic transformation was applied because the raw ratios exhibited markedly right-skewed distributions. This transformation normalized the distributions, stabilized variance, and satisfied the log-linearity assumption of the Cox proportional hazards model (22).

### Statistical analysis

Continuous variables were expressed as mean ± standard deviation (SD) or median (interquartile range, IQR), and categorical variables as frequencies (percentages). Intergroup comparisons were performed using Student’s t-test, Mann–Whitney U test, or chi-square test, stratified by cohort and 30-day survival status.

Survival analysis was conducted as follows. First, Kaplan–Meier survival curves were plotted for each inflammatory ratio (grouped by tertiles) in both cohorts and compared using the log-rank test. Second, multivariable Cox proportional hazards regression models were constructed to evaluate the independent associations of each inflammatory ratio with 30-day mortality, analyzed both as continuous variables and as categorical variables grouped by tertiles. Three progressively adjusted models were established: Model 1, unadjusted; Model 2, adjusted for demographic characteristics (age, sex, and weight); and Model 3, further adjusted for laboratory parameters, vital signs, severity scores, comorbidities, and treatment interventions based on Model 2. To avoid overfitting in the smaller external cohort (*n* = 268, 56 events), Model 3 was restricted to key clinical confounders according to the events-per-variable (EPV) principle. With 56 observed events, we conservatively limited the model to 5 covariates (EPV = 11.2). These 5 variables were selected based on their established prognostic significance in RHD and critical care literature: age and gender, weight, SOFA score, and valve surgery (23–25). Results were reported as hazard ratios (HR) and 95% confidence intervals (CI). Restricted cubic spline (RCS) analysis was used to examine nonlinear dose–response relationships between each continuous inflammatory ratio and mortality risk.

To evaluate the predictive discrimination of each biomarker for 30-day mortality, time-dependent receiver operating characteristic (ROC) curves were constructed. Additionally, to assess the incremental predictive value of inflammatory ratios beyond traditional critical illness scores, a baseline prediction model comprising only disease severity scores was established, and each inflammatory ratio was subsequently added individually. The integrated discrimination improvement (IDI) index was calculated to quantify the enhancement in predictive performance afforded by each ratio.

To evaluate whether the observed associations were influenced by patients with hyperacute fatal events or underlying malignancy, we conducted two sensitivity analyses in the MIMIC-IV cohort. In the first analysis, patients who died within 7 days of ICU admission were excluded. In the second analysis, patients with documented malignant tumors were excluded. For each restricted cohort, we refitted the three Cox regression models using identical covariate specifications as the primary analysis.

All statistical analyses were performed using R software (version 4.3.0). A two-sided *p*-value < 0.05 was considered statistically significant.

## Results

### Baseline characteristics

A total of 764 RHD patients were included, comprising 496 in the MIMIC and 268 in the validation cohort. In the MIMIC cohort (Table 1), non-survivors were significantly older and had higher disease severity scores, alongside elevated potassium, anion gap, urea nitrogen, creatinine, neutrophil, and RDW, as well as reduced albumin and lymphocyte levels. Notably, NAR and RAR were significantly higher in non-survivors, while LAR was significantly lower. Non-survivors also exhibited lower SBP and DBP, higher rates of malignant tumors, kidney disease, sepsis, and antibiotic use, and lower valve surgery rates. Similarly, in the validation cohort (Table 2), non-survivors presented with higher disease severity scores and elevated anion gap, urea nitrogen, creatinine, potassium, RDW, and neutrophil, as well as lower lymphocyte levels. Consistently, NAR and RAR remained significantly higher in non-survivors, and LAR significantly lower. Non-survivors in this cohort also had lower blood pressure, higher rates of congestive heart failure and antibiotic use, and lower valve surgery rates.

**Table 1:** Baseline characteristics of RHD patients grouped according to 30-day mortality in MIMIC cohort.

Variables	Total	Survivor	Non-survivor	P
	(*n* = 496)	(*n* = 385)	(*n* = 111)	
Age, years	75.97 ± 13.93	75.15 ± 14.48	78.81 ± 11.43	0.006
Gender, %				0.425
Female	274 (55.24)	209 (54.29)	65 (58.56)	
Male	222 (44.76)	176 (45.71)	46 (41.44)	
Weight, Kg	75.39 ± 19.82	75.75 ± 19.49	74.15 ± 20.96	0.453
Sodium, mmol/L	136.05 ± 5.45	135.99 ± 5.48	136.23 ± 5.35	0.684
Potassium, mmol/L	3.98 ± 0.61	3.95 ± 0.57	4.10 ± 0.73	0.047
Calcium, mg/dL	8.24 ± 0.83	8.28 ± 0.84	8.11 ± 0.81	0.06
Anion gap, mmol/L	13.74 ± 3.77	13.21 ± 3.30	15.59 ± 4.63	<0.001
Urea nitrogen, mg/dL	34.96 ± 24.83	32.07 ± 22.07	44.99 ± 30.72	<0.001
Glucose, mg/dL	115.28 ± 40.17	113.61 ± 36.84	121.07 ± 49.80	0.145
Creatinine, mg/dL	1.73 ± 1.62	1.62 ± 1.63	2.13 ± 1.54	0.004
Albumin g/dL	3.25 ± 0.59	3.29 ± 0.59	3.14 ± 0.57	0.017
Platelet, K/μL	175.63 ± 93.45	175.52 ± 94.57	176.03 ± 89.86	0.96
RDW, %	16.13 ± 2.65	15.81 ± 2.44	17.23 ± 3.05	<0.001
Neutrophil, K/uL	10.13 ± 6.48	9.74 ± 6.17	11.48 ± 7.35	0.012
Monocyte, K/uL	0.71 ± 0.59	0.70 ± 0.62	0.72 ± 0.44	0.721
Lymphocyte, K/uL	1.23 ± 1.03	1.31 ± 1.09	0.95 ± 0.74	<0.001
NAR	0.98 ± 0.66	0.93 ± 0.67	1.16 ± 0.62	0.001
LAR	−1.18 ± 0.68	−1.13 ± 0.68	−1.38 ± 0.64	<0.001
MAR	−1.74 ± 0.73	−1.76 ± 0.72	−1.67 ± 0.75	0.259
PAR	3.87 ± 0.59	3.86 ± 0.57	3.90 ± 0.64	0.479
RAR	1.61 ± 0.27	1.58 ± 0.25	1.71 ± 0.29	<0.001
Respiratory rate, minute	12.92 ± 3.77	12.92 ± 3.77	12.94 ± 3.78	0.961
SBP, mmHg	86.70 ± 16.49	88.50 ± 15.64	80.46 ± 17.87	<0.001
DBP, mmHg	43.66 ± 11.08	44.40 ± 10.82	41.09 ± 11.62	0.005
Heart rate, minute	70.27 ± 16.84	69.63 ± 16.20	72.49 ± 18.81	0.115
SOFA	5.90 ± 3.51	5.42 ± 3.26	7.59 ± 3.83	<0.001
SAPS II	43.07 ± 13.13	40.66 ± 11.38	51.43 ± 15.24	<0.001
OASIS	34.00 ± 8.48	32.80 ± 7.93	38.14 ± 9.04	<0.001
Hypertension, %				0.165
No	368 (74.19)	280 (72.73)	88 (79.28)	
Yes	128 (25.81)	105 (27.27)	23 (20.72)	
Diabetes, %				0.283
No	325 (65.52)	257 (66.75)	68 (61.26)	
Yes	171 (34.48)	128 (33.25)	43 (38.74)	
Malignant tumor, %				0.035
No	457 (92.14)	360 (93.51)	97 (87.39)	
Yes	39 (7.86)	25 (6.49)	14 (12.61)	
Kidney disease, %				0.027
No	291 (58.67)	236 (61.30)	55 (49.55)	
Yes	205 (41.33)	149 (38.70)	56 (50.45)	
Sepsis, %				<0.001
No	190 (38.31)	172 (44.68)	18 (16.22)	
Yes	306 (61.69)	213 (55.32)	93 (83.78)	
Chronic pulmonary disease, %				0.359
No	322 (64.92)	254 (65.97)	68 (61.26)	
Yes	174 (35.08)	131 (34.03)	43 (38.74)	
Congestive heart failure, (%)				0.377
No	109 (21.98)	88 (22.86)	21 (18.92)	
Yes	387 (78.02)	297 (77.14)	90 (81.08)	
Drug antibiotic used, %				<0.001
No	128 (25.81)	117 (30.39)	11 (9.91)	
Yes	368 (74.19)	268 (69.61)	100 (90.09)	
Valve surgery, %				0.003
No	439 (88.51)	332 (86.23)	107 (96.40)	
Yes	57 (11.49)	53 (13.77)	4 (3.60)	
Infusion of albumin, %				0.060
No	368 (74.19)	278 (72.21)	90 (81.08)	
Yes	128 (25.81)	107 (27.79)	21(18.92)	

RDW, red blood cell volume distribution width; PAR, platelet to albumin ratio; LAR, lymphocyte to albumin ratio; MAR, monocyte to albumin ratio; NAR, neutrophil to albumin ratio; RAR, red cell distribution width to albumin ratio; SBP, systolic blood pressure; DBP, diastolic blood pressure; SOFA, sequential organ failure assessment; SAPS II, simplified acute physiology; OASIS, oxford acute severity of illness score.

All values are expressed as a proportion (%) or mean ± standard deviation (SD).

**Table 2:** Baseline characteristics of RHD patients grouped according to 30-day mortality in external validation cohort.

Variables	Total (*n* = 268)	Survivor (*n* = 212)	Non-survivor (*n* = 56)	*P*
Age, years	74.73 ± 14.55	73.86 ± 15.05	77.99 ± 12.03	0.059
Gender, %				0.802
Female	138 (51.49)	110 (51.89)	28 (50.00)	
Male	130 (48.51)	102 (48.11)	28 (50.00)	
Weight, Kg	76.46 ± 19.64	76.36 ± 19.00	76.84 ± 22.05	0.871
Sodium, mmol/L	136.29 ± 5.88	136.17 ± 5.86	136.77 ± 5.98	0.496
Potassium, mmol/L	4.00 ± 0.59	3.96 ± 0.57	4.16 ± 0.65	0.022
Calcium, mg/dL	8.25 ± 0.91	8.28 ± 0.92	8.12 ± 0.85	0.228
Anion gap, mmol/L	13.74 ± 3.61	13.20 ± 3.22	15.77 ± 4.24	<0.001
Urea nitrogen, mg/dL	34.37 ± 23.42	31.58 ± 20.51	44.95 ± 30.10	0.003
Glucose, mg/dL	117.57 ± 43.12	115.39 ± 38.79	125.84 ± 56.28	0.194
Creatinine, mg/dL	1.71 ± 1.62	1.57 ± 1.61	2.24 ± 1.55	0.006
Albumin, g/dL	3.22 ± 0.61	3.25 ± 0.60	3.10 ± 0.62	0.096
Platelet, K/μL	168.52 ± 84.83	165.02 ± 84.14	181.75 ± 86.87	0.19
RDW, %	16.12 ± 2.77	15.74 ± 2.46	17.57 ± 3.38	<0.001
Neutrophil, K/uL	9.85 ± 6.39	9.42 ± 5.81	11.47 ± 8.07	0.032
Monocyte, K/uL	0.65 ± 0.40	0.64 ± 0.39	0.68 ± 0.45	0.472
Lymphocyte, K/uL	1.31 ± 1.15	1.42 ± 1.22	0.91 ± 0.74	0.004
NAR	0.96 ± 0.68	0.91 ± 0.68	1.15 ± 0.67	0.018
LAR	−1.11 ± 0.69	−1.03 ± 0.68	−1.41 ± 0.65	<0.001
MAR	−1.80 ± 0.74	−1.82 ± 0.72	−1.75 ± 0.83	0.577
PAR	3.85 ± 0.57	3.82 ± 0.56	3.97 ± 0.60	0.062
RAR	1.62 ± 0.27	1.58 ± 0.26	1.74 ± 0.30	<0.001
Respiratory rate, minute	13.13 ± 3.87	13.10 ± 3.87	13.25 ± 3.89	0.802
SBP, mmHg	87.03 ± 16.21	88.85 ± 15.73	80.14 ± 16.30	<0.001
DBP, mmHg	43.75 ± 11.30	44.79 ± 11.31	39.82 ± 10.44	0.003
Heart rate, minute	69.98 ± 17.65	70.13 ± 17.20	69.41 ± 19.42	0.786
SOFA	6.01 ± 3.45	5.48 ± 3.20	8.04 ± 3.65	<0.001
SAPS II	43.02 ± 13.63	40.43 ± 11.74	52.80 ± 15.82	<0.001
OASIS	33.77 ± 8.44	32.62 ± 7.81	38.11 ± 9.35	<0.001
Hypertension, %				0.268
No	200 (74.63)	155 (73.11)	45 (80.36)	
Yes	68 (25.37)	57 (26.89)	11 (19.64)	
Diabetes, %				0.471
No	169 (63.06)	136 (64.15)	33 (58.93)	
Yes	99 (36.94)	76 (35.85)	23 (41.07)	
Congestive heart failure, %				0.046
No	60 (22.39)	53 (25.00)	7 (12.50)	
Yes	208 (77.61)	159 (75.00)	49 (87.50)	
Drug antibiotic used, %				0.011
No	69 (25.75)	62 (29.25)	7 (12.50)	
Yes	199 (74.25)	150 (70.75)	49 (87.50)	
Valve surgery, %				0.008
No	236 (88.06)	181 (85.38)	55 (98.21)	
Yes	32 (11.94)	31 (14.62)	1 (1.79)	
Infusion of albumin, %				0.134
No	194 (72.39)	149 (70.28)	45 (80.36)	
Yes	74 (27.61)	63 (29.72)	11 (19.64)	

RDW, red blood cell volume distribution width; PAR, platelet to albumin ratio; LAR, lymphocyte to albumin ratio; MAR, monocyte to albumin ratio; NAR, neutrophil to albumin ratio; RAR, red cell distribution width to albumin ratio; SBP, systolic blood pressure; DBP, diastolic blood pressure; SOFA, sequential organ failure assessment; SAPS II, simplified acute physiology; OASIS, oxford acute severity of illness score.

All values are expressed as a proportion (%) or mean ± standard deviation (SD).

No significant differences were observed between the two cohorts in terms of demographic characteristics, laboratory parameters, inflammatory ratios, vital signs, disease severity scores, comorbidities, or therapeutic interventions (all *p* > 0.05, Supplementary Table S1).

### Kaplan–Meier survival analysis

**Figure 2:**
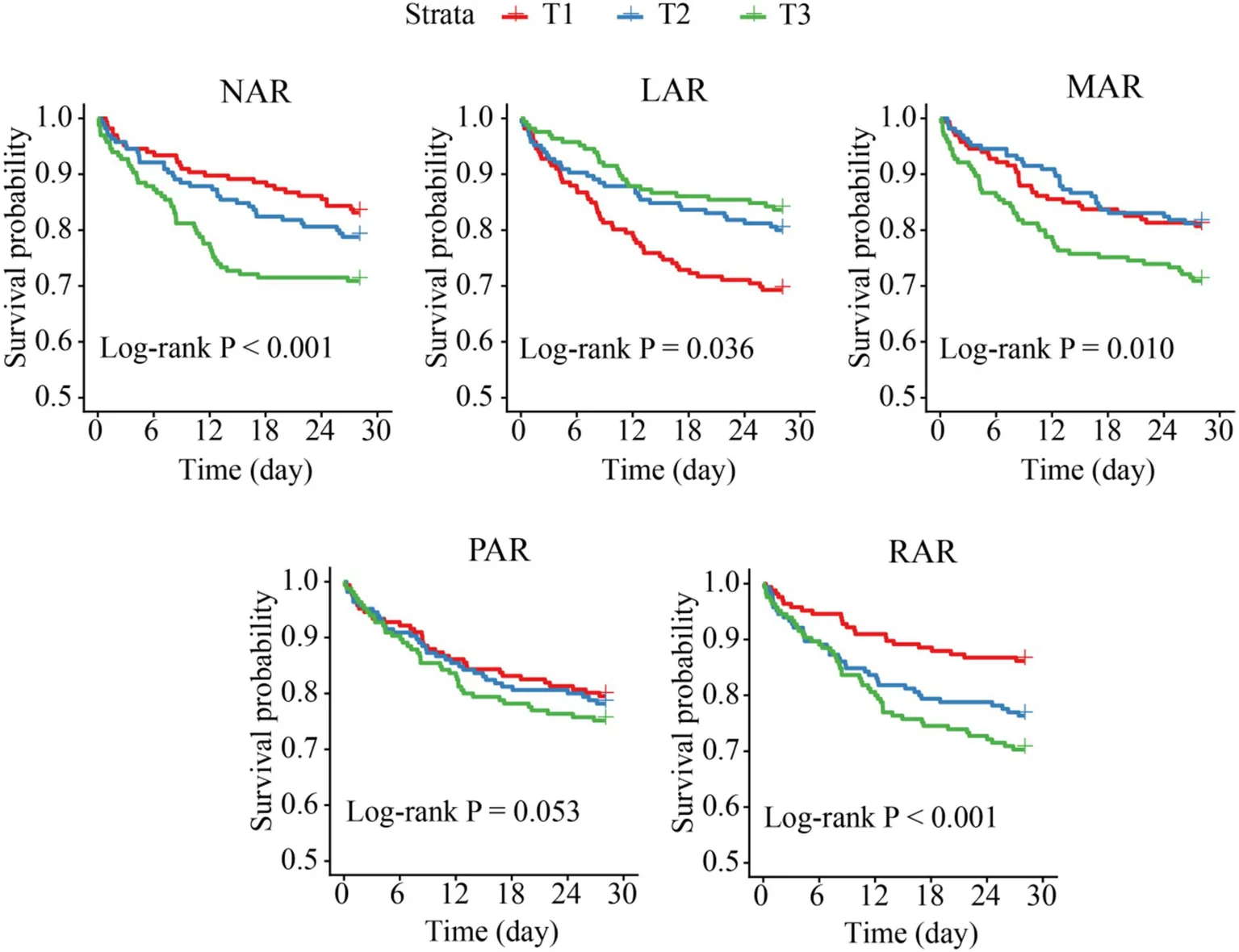
Kaplan–Meier survival curves for 30-day mortality according to tertiles of albumin-related inflammatory biomarkers in patients with RHD (MIMIC-IV cohort).

**Figure 3:**
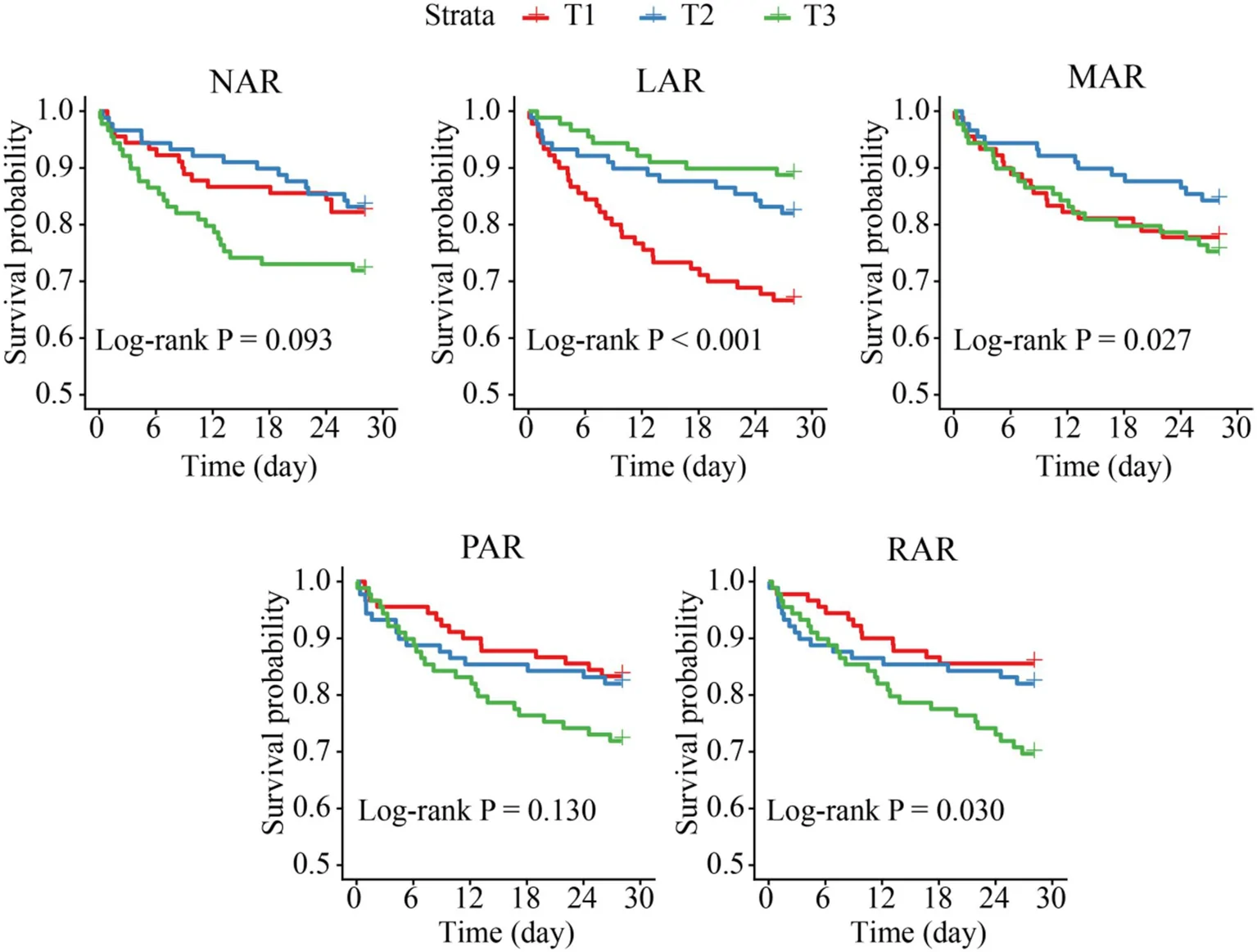
Kaplan–Meier survival curves for 30-day mortality according to tertiles of albumin-related inflammatory biomarkers in patients with RHD (validation cohort).

Kaplan–Meier survival analysis revealed distinct prognostic patterns across biomarkers in both cohorts (Figures 2, 3). In the MIMIC-IV cohort, higher tertiles of NAR (log-rank *p* < 0.001), MAR (*p* = 0.010), and RAR (p < 0.001), and lower tertiles of LAR (*p* = 0.036), were significantly associated with reduced 30-day survival, whereas PAR showed no significant association (*p* = 0.053). In the validation cohort, LAR (*p* < 0.001), MAR (*p* = 0.027), and RAR (*p* = 0.030) remained significant, with lower LAR tertiles, higher MAR and RAR tertiles predicting worse outcomes. NAR (*p* = 0.093) and PAR (*p* = 0.130) did not achieve statistical significance in this cohort.

### Cox proportional hazards regression analysis

Multivariable Cox proportional hazards regression analysis was performed to evaluate the independent associations of albumin-related inflammatory biomarkers with 30-day mortality in both cohorts (Table 3). In the fully adjusted Model 3, RAR remained significantly associated with mortality as a continuous variable in both the MIMIC-IV cohort (HR 4.12, 95% CI 1.79–9.49; *p* < 0.001) and the validation cohort (HR 3.20, 95% CI 1.25–8.23; *p* = 0.016). Furthermore, the highest tertile of RAR conferred significantly elevated mortality risk in both cohorts (MIMIC-IV: HR 1.93, 95% CI 1.11–3.33, *p* = 0.019; validation: HR 1.79, 95% CI 1.18–2.62, *p* = 0.037). LAR demonstrated a protective effect exclusively in the validation cohort, with both continuous (HR 0.61, 95% CI 0.43–0.88; *p* = 0.007) and highest tertile (HR 0.36, 95% CI 0.18–0.74; *p* = 0.005) analyses reaching statistical significance. NAR showed cohort-specific significance as a continuous variable in the MIMIC-IV cohort (HR 1.50, 95% CI 1.09–2.07; *p* = 0.001) but not in the validation cohort (*p* = 0.075). MAR exhibited limited prognostic value, with significance observed only for the highest tertile in the MIMIC-IV cohort (HR 1.60, 95% CI 1.01–2.53; *p* = 0.047). PAR demonstrated no significant independent association with 30-day mortality in either cohort.

**Table 3:** Multivariate COX regression analysis of albumin-related inflammatory biomarkers and 30-day mortality in RHD patients.

Indices		Model 1 HR (95% CI)	*P*	Model 2 HR (95% CI)	*P*	Model 3 HR (95% CI)	*P*
MIMIC cohort							
PAR	Continuous	1.12 (0.81–1.56)	0.488	1.13 (0.81–1.57)	0.490	1.23 (0.86–1.76)	0.263
Tertiles	T1	1.00		1.00		1.00	
	T2	1.07 (0.67–1.72)	0.762	1.05 (0.66–1.68)	0.842	1.28 (0.78–2.09)	0.328
	T3	1.25 (0.79–1.97)	0.334	1.26 (0.79–1.99)	0.332	1.61 (0.97–2.68)	0.067
NAR	Continuous	1.63 (1.23–2.17)	<0.001	1.75 (1.31–2.35)	<0.001	1.50 (1.09–2.07)	0.001
Tertiles	T1	1.00		1.00		1.00	
	T2	1.29 (0.79–2.13)	0.310	1.32 (0.80–2.17)	0.273	0.92 (0.55–1.54)	0.748
	T3	1.92 (1.20–3.06)	0.006	2.14 (1.34–3.44)	0.002	1.34 (0.82–2.20)	0.247
LAR	Continuous	0.62 (0.47–0.81)	<0.001	0.64 (0.48–0.84)	0.001	0.85 (0.65–1.10)	0.219
Tertiles	T1	1.00		1.00		1.00	
	T2	0.61 (0.39–0.95)	0.027	0.61 (0.40–0.95)	0.030	0.77 (0.49–1.23)	0.278
	T3	0.48 (0.30–0.77)	0.002	0.52 (0.33–0.84)	0.007	0.62 (0.38–1.02)	0.060
MAR	Continuous	1.17 (0.90–1.52)	0.245	1.17 (0.90–1.52)	0.251	1.17 (0.91–1.50)	0.214
Tertiles	T1	1.00		1.00		1.00	
	T2	0.96 (0.58–1.57)	0.859	0.93 (0.57–1.52)	0.769	0.96 (0.58–1.58)	0.866
	T3	1.62 (1.04–2.54)	0.034	1.62 (1.04–2.54)	0.034	1.60 (1.01–2.53)	0.047
RAR	Continuous	4.91 (2.49–9.66)	<0.001	5.73 (2.91–11.29)	<0.001	4.12 (1.79–9.49)	<0.001
Tertiles	T1	1.00		1.00		1.00	
	T2	1.82 (1.09–3.05)	0.023	1.80 (1.07–3.01)	0.026	1.49 (0.87–2.56)	0.147
	T3	2.35 (1.43–3.85)	<0.001	2.62 (1.59–4.32)	<0.001	1.93 (1.11–3.33)	0.019
Validation cohort							
PAR	Continuous	1.32 (0.88–1.96)	0.358	1.41 (0.91–2.06)	0.178	1.53 (0.97–1.97)	0.072
Tertiles	T1	1.00		1.00		1.00	
	T2	1.09 (0.65–1.87)	0.753	1.06 (0.52–2.11)	0.816	1.32 (0.66–2.82)	0.370
	T3	1.45 (0.85–2.43)	0.270	1.52 (0.88–2.86)	0.137	1.79 (0.98–3.48)	0.067
NAR	Continuous	1.62 (1.09–2.40)	0.016	1.74 (1.16–2.62)	0.007	1.45 (0.96–2.20)	0.075
Tertiles	T1	1.00		1.00		1.00	
	T2	0.93 (0.46–1.87)	0.830	0.93 (0.46–1.87)	0.829	0.86 (0.42–1.75)	0.674
	T3	1.71 (0.91–3.21)	0.093	1.87 (0.99–3.54)	0.055	1.43 (0.75–2.74)	0.282
LAR	Continuous	0.50 (0.34–0.72)	<0.001	0.53 (0.36–0.78)	0.001	0.61 (0.43–0.88)	0.007
Tertiles	T1	1.00		1.00		1.00	
	T2	0.49 (0.26–0.89)	0.020	0.52 (0.28–0.97)	0.039	0.59 (0.32–1.10)	0.098
	T3	0.29 (0.14–0.60)	<0.001	0.32 (0.16–0.67)	0.002	0.36 (0.18–0.74)	0.005
MAR	Continuous	1.10 (0.76–1.60)	0.602	1.10 (0.76–1.59)	0.603	1.14 (0.79–1.63)	0.486
Tertiles	T1	1.00		1.00		1.00	
	T2	0.67 (0.34–1.32)	0.246	0.65 (0.33–1.29)	0.221	0.73 (0.37–1.46)	0.377
	T3	1.11 (0.61–2.04)	0.728	1.12 (0.61–2.05)	0.720	1.19 (0.64–2.21)	0.583
RAR	Continuous	5.68 (2.30–14.00)	<0.001	7.09 (2.85–17.65)	<0.001	3.20 (1.25–8.23)	0.016
Tertiles	T1	1.00		1.00		1.00	
	T2	1.60 (1.02–2.90)	0.086	1.76 (0.91–2.64)	0.083	1.24 (0.64–2.71)	0.525
	T3	2.25 (1.16–4.36)	0.016	2.38 (1.34–5.15)	0.005	1.79 (1.18–2.62)	0.037

Model 1: no covariates were adjusted.

Model 2: age, gender, and weight were adjusted.

Model 3: MIMIC cohort: age, weight, SBP, DBP, anion gap, urea nitrogen, creatinine, potassium, SOFA, SAPS II, OASIS, sepsis, drug antibiotic used, and valve surgery were adjusted. The validation cohort: age, gender, weight, SOFA score, and valve surgery were adjusted.

### Dose–response relationships

**Figure 4:**
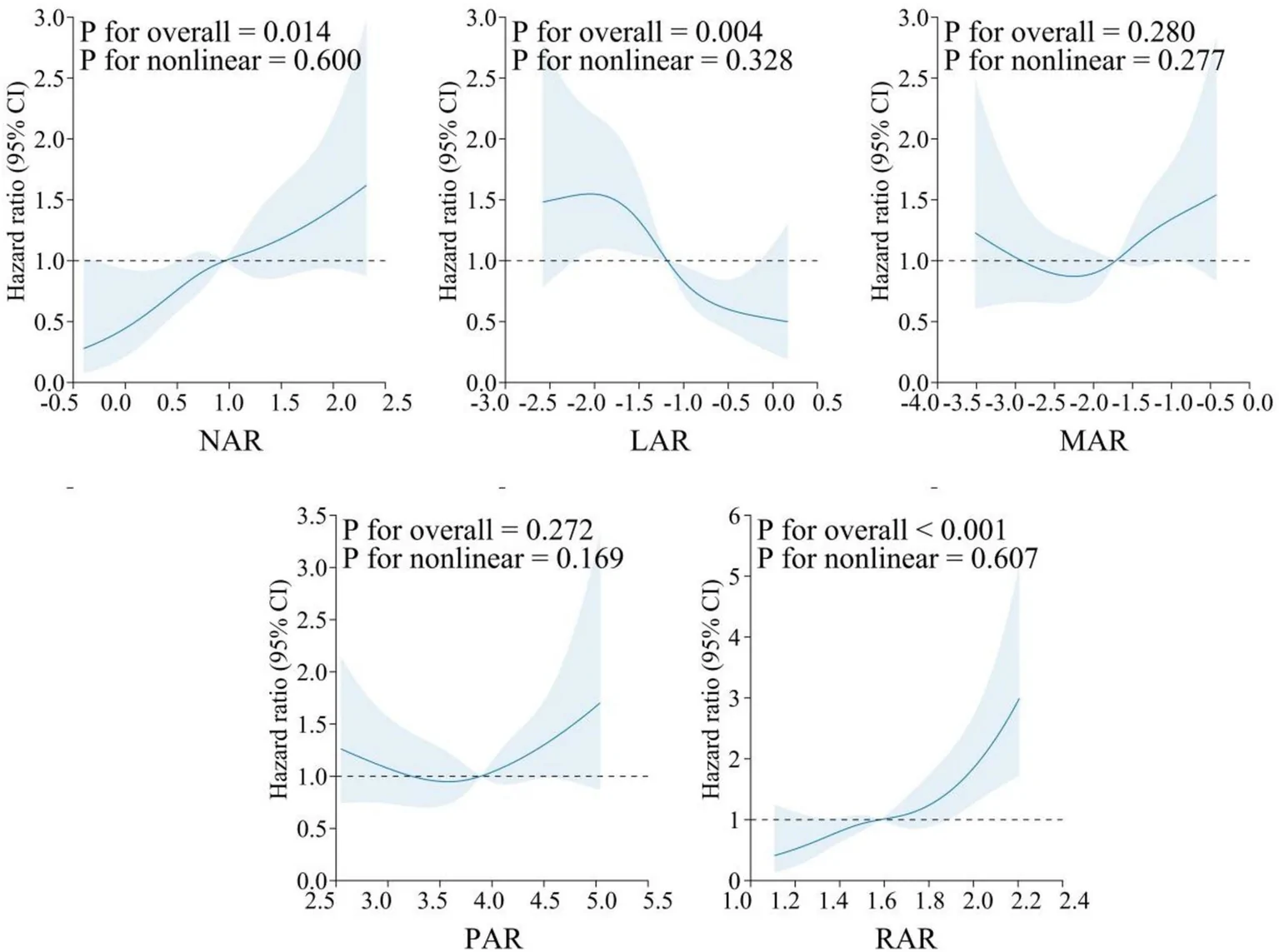
Dose–response relationships between albumin-related inflammatory biomarkers and 30-day mortality risk in patients with RHD (MIMIC-IV cohort). Solid lines represent hazard ratios, and shaded areas indicate 95% confidence intervals.

**Figure 5:**
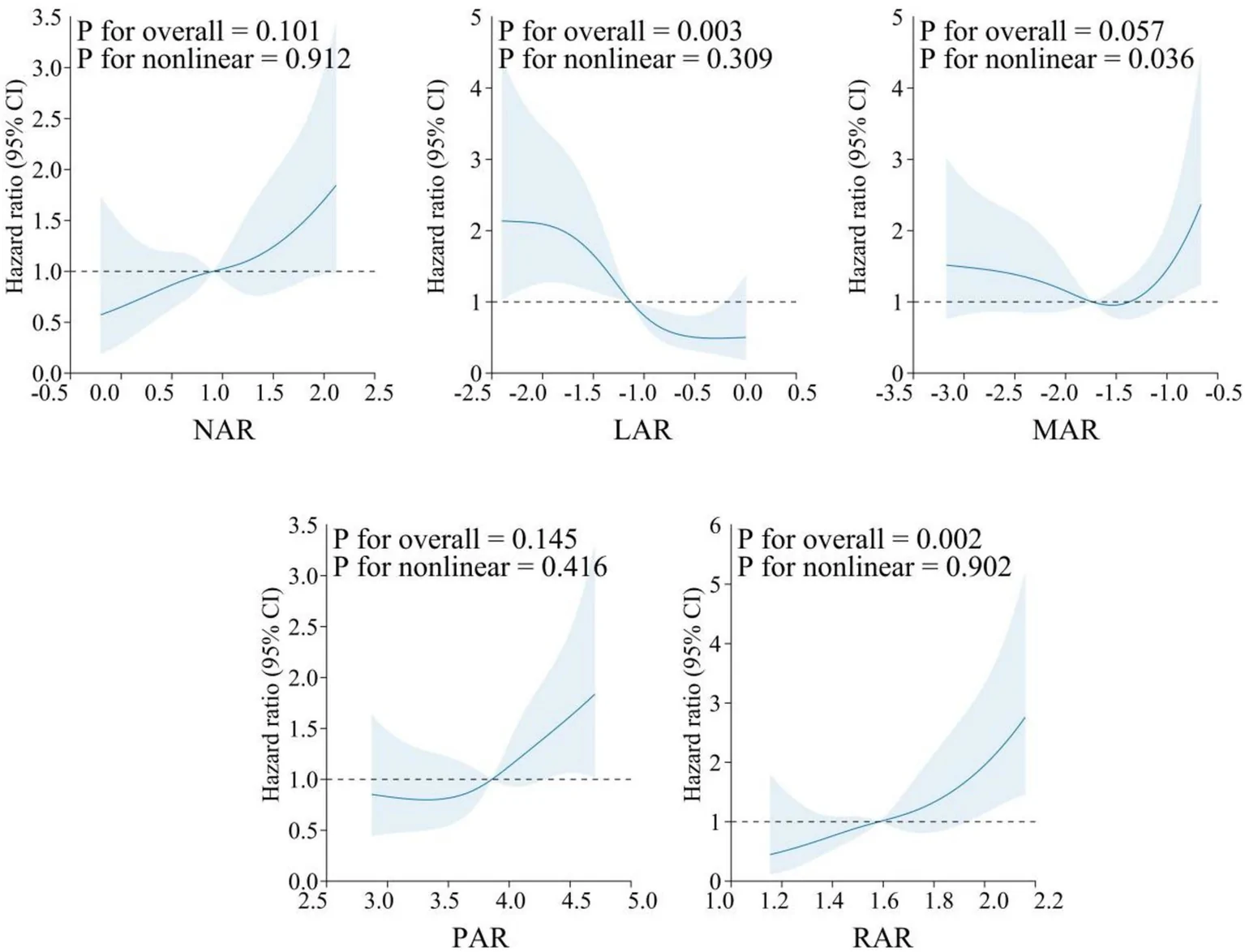
Dose–response relationships between albumin-related inflammatory biomarkers and 30-day mortality risk in patients with RHD (validation cohort). Solid lines represent hazard ratios, and shaded areas indicate 95% confidence intervals.

RCS analysis revealed distinct dose–response patterns across biomarkers in both cohorts (Figures 4, 5). In the MIMIC-IV cohort, RAR demonstrated a significant positive linear association with 30-day mortality (P for overall < 0.001; P for nonlinear = 0.607), with HR increasing progressively across the observed range. NAR also showed a significant positive linear relationship (P for overall = 0.014; P for nonlinear = 0.600), whereas LAR exhibited a significant inverse linear association (P for overall = 0.004; P for nonlinear = 0.328). MAR and PAR showed no significant overall associations (*p* = 0.280 and *p* = 0.272, respectively). In the validation cohort, RAR again displayed a significant positive linear association (P for overall = 0.002; P for nonlinear = 0.902), and LAR showed a significant inverse linear relationship (P for overall = 0.003; P for nonlinear = 0.309). NAR, MAR, and PAR did not achieve statistical significance in this cohort.

### Predictive performance of inflammatory biomarkers

**Figure 6:**
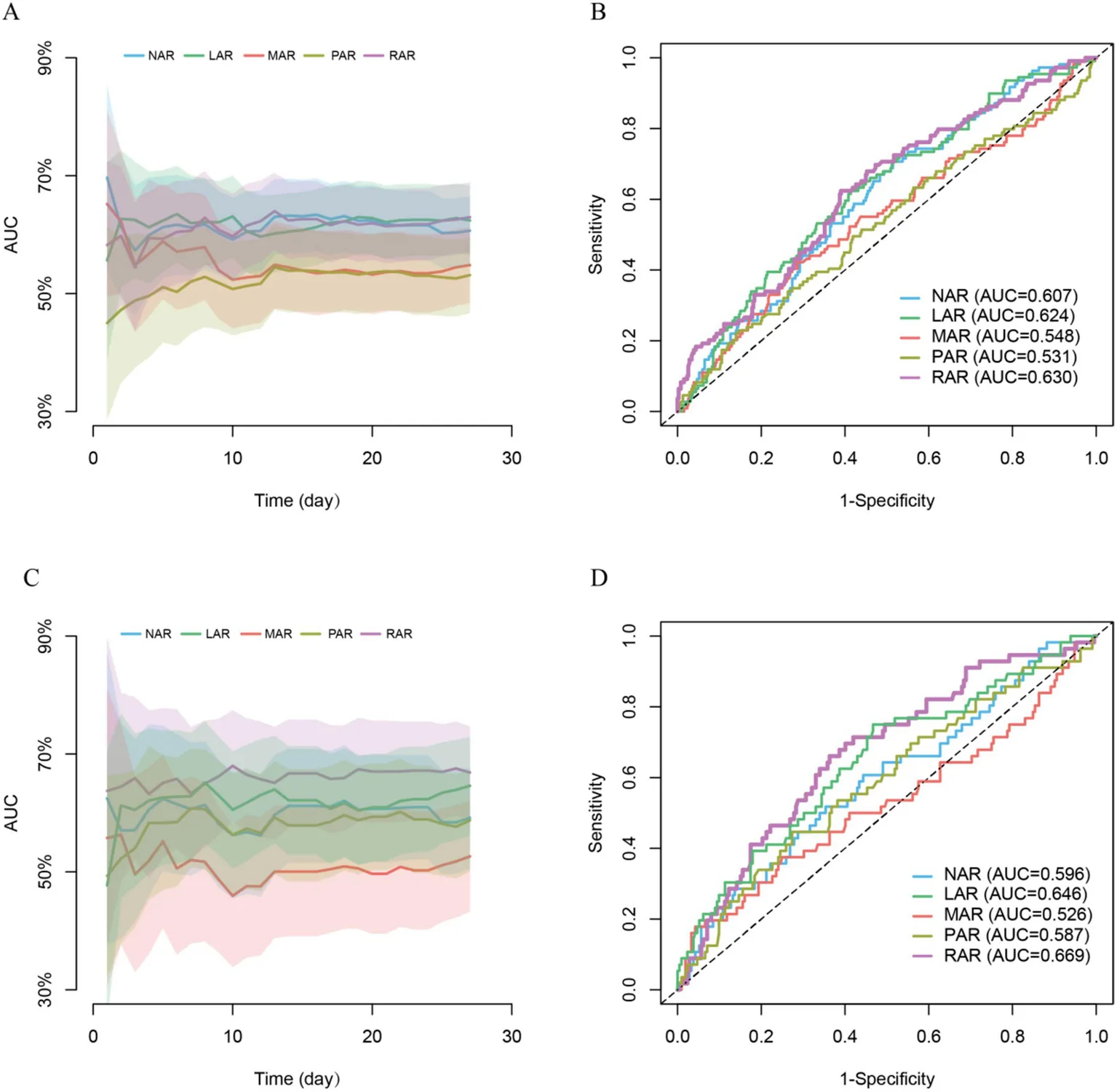
Predictive performance of albumin-related inflammatory biomarkers for 30-day mortality in patients with RHD. Time-dependent AUC curves and ROC curves in the MIMIC-IV cohort **(A,B)** and validation cohort **(C,D)**. Shaded areas in **A** and **C** represent 95% confidence intervals.

Time-dependent AUC analysis revealed that the discriminative abilities of the five albumin-related inflammatory biomarkers for mortality risk varied across time points in patients with rheumatic heart disease. Overall, NAR, LAR, and RAR demonstrated relatively strong discriminative performance throughout the follow-up period in both the MIMIC-IV and validation cohorts. Furthermore, time-dependent ROC curves at 30 days corroborated these findings: RAR achieved the highest AUC values of 0.630 in the MIMIC-IV cohort and 0.669 in the validation cohort, surpassing LAR (0.624 and 0.646), NAR (0.607 and 0.596), PAR (0.531 and 0.587), and MAR (0.548 and 0.526) (Figure 6).

### Incremental predictive value of albumin-related inflammatory indices

The IDI analysis demonstrated that RAR was the only biomarker to provide significant incremental predictive value beyond all three established severity scores in both cohorts (Table 4). In the MIMIC cohort, RAR provided the most substantial incremental value across all three scores, while LAR also showed modest improvement for OASIS. In the validation cohort, RAR significantly improved the discrimination ability for all three scoring systems (IDI values of 0.056, 0.039, and 0.047, respectively; all *p* < 0.05), whereas other albumin-related inflammatory indices did not enhance the predictive value of disease severity scores for prognosis.

**Table 4:** Incremental value of adding albumin-related inflammatory ratios to established severity scores for predicting 30-day mortality in RHD.

Indices	SOFA IDI (95% CI)	*P*	SAPSII IDI (95% CI)	*P*	OASIS IDI (95% CI)	*P*
MIMIC cohort						
PAR	0.019 (0.003, 0.053)	<0.001	0.004 (−0.004, 0.023)	0.319	0.003 (−0.001, 0.021)	0.292
LAR	0.015 (0.000, 0.045)	0.033	0.011 (−0.003, 0.035)	0.100	0.013 (0.000, 0.044)	0.047
MAR	0.007 (−0.001, 0.027)	0.086	0.005 (−0.002, 0.023)	0.166	0.001 (−0.002, 0.015)	0.545
NAR	0.013 (0.000,0.041)	0.047	0.013 (0.000, 0.038)	0.060	0.011 (−0.001, 0.038)	0.106
RAR	0.022 (0.006, 0.055)	<0.001	0.016 (0.005, 0.038)	0.006	0.034 (0.011, 0.073)	<0.001
Validation cohort						
PAR	0.022 (−0.003, 0.084)	0.093	0.016 (−0.004, 0.058)	0.126	0.010 (−0.003, 0.047)	0.206
LAR	0.042 (0.000, 0.093)	0.053	0.028 (−0.010, 0.082)	0.106	0.015 (−0.010, 0.061)	0.179
MAR	0.010 (−0.003, 0.065)	0.259	0.003 (−0.005, 0.041)	0.445	0.000 (−0.001, 0.027)	0.292
NAR	0.007 (−0.002, 0.051)	0.213	0.007 (−0.002, 0.042)	0.173	0.011 (−0.001, 0.058)	0.086
RAR	0.056 (0.006, 0.111)	0.013	0.039 (0.002, 0.097)	0.027	0.047 (0.009, 0.116)	0.007

IDI, integrated discrimination improvement; CI, confidence interval; SOFA, sequential organ failure assessment; SAPSII, simplified acute physiology score II; OASIS, oxford acute severity of illness score; PAR, platelet to albumin ratio; LAR, lymphocyte to albumin ratio; MAR, monocyte to albumin ratio; NAR, neutrophil to albumin ratio; RAR, red cell distribution width to albumin ratio. IDI values are presented as an estimate (95% CI).

### Sensitivity analysis

To evaluate whether the observed associations were driven by patients with hyperacute fatal events or underlying malignancy, we performed two sensitivity analyses in the MIMIC-IV cohort (Table 5). After excluding 44 patients who died within 7 days of ICU admission (remaining *n* = 452; 67 non-survivors and 385 survivors), RAR remained significantly associated with 30-day mortality in the fully adjusted Model 3 as a continuous variable (HR 4.64, 95% CI 1.49–14.42; *p* = 0.008) and across tertiles (T3: HR 2.03, 95% CI 1.03–3.99; *p* = 0.040). Similarly, after excluding 39 patients with documented malignant tumors (remaining *n* = 457; 97 non-survivors and 360 survivors), the association between RAR and mortality remained robust in Model 3 (continuous: HR 3.66, 95% CI 1.42–9.40; *p* = 0.007; T3: HR 1.87, 95% CI 1.04–3.36; *p* = 0.037).

**Table 5:** Cox regression sensitivity analysis excluding early deaths and malignancy (MIMIC-IV cohort).

Indices		Model 1 HR (95% CI)	*P*	Model 2 HR (95% CI)	*P*	Model 3 HR (95% CI)	*P*
Exclude patients who died within 7 days							
PAR	Continuous	1.19 (0.77–1.83)	0.430	1.20 (0.77–1.87)	0.414	1.49 (0.93–2.41)	0.099
Tertiles	T1	1.00		1.00		1.00	
	T2	1.12 (0.62–2.02)	0.715	1.09 (0.60–1.98)	0.766	1.44 (0.78–2.67)	0.245
	T3	1.16 (0.64–2.09)	0.626	1.16 (0.64–2.12)	0.620	1.69 (0.87–3.27)	0.119
NAR	Continuous	1.66 (1.16–2.39)	0.006	1.77 (1.22–2.56)	0.003	1.53 (1.02–2.28)	0.039
Tertiles	T1	1.00		1.00		1.00	
	T2	1.30 (0.68–2.46)	0.427	1.32 (0.70–2.50)	0.396	0.97 (0.49–1.89)	0.918
	T3	2.00 (1.10–3.64)	0.023	2.20 (1.20–4.05)	0.011	1.46 (0.77–2.76)	0.247
LAR	Continuous	0.63 (0.45–0.89)	0.009	0.65 (0.45–0.92)	0.015	0.83 (0.59–1.17)	0.287
Tertiles	T1	1.00		1.00		1.00	
	T2	0.55 (0.30–0.98)	0.042	0.54 (0.30–0.98)	0.042	0.67 (0.36–1.23)	0.195
	T3	0.56 (0.31–0.99)	0.046	0.59 (0.33–1.07)	0.080	0.71 (0.39–1.32)	0.280
MAR	Continuous	1.20 (0.85–1.68)	0.307	1.19 (0.85–1.67)	0.317	1.20 (0.86–1.66)	0.276
Tertiles	T1	1.00		1.00		1.00	
	T2	1.03 (0.55–1.93)	0.927	1.00 (0.53–1.88)	0.995	1.09 (0.58–2.07)	0.786
	T3	1.63 (0.91–2.91)	0.102	1.62 (0.90–2.90)	0.106	1.65 (0.90–3.00)	0.104
RAR	Continuous	4.88 (2.01–11.83)	<0.001	5.78 (2.36–14.13)	<0.001	4.64 (1.49–14.42)	0.008
Tertiles	T1	1.00		1.00		1.00	
	T2	1.40 (0.71–2.75)	0.332	1.38 (0.70–2.73)	0.346	1.12 (0.55–2.27)	0.752
	T3	2.53 (1.37–4.65)	0.003	2.83 (1.52–5.25)	<0.001	2.03 (1.03–3.99)	0.040
Exclude patients with malignant tumors							
PAR	Continuous	1.03 (0.72–1.49)	0.857	1.03 (0.71–1.50)	0.856	1.10 (0.75–1.62)	0.632
Tertiles	T1	1.00		1.00		1.00	
	T2	1.13 (0.69–1.86)	0.623	1.11 (0.68–1.83)	0.670	1.36 (0.80–2.29)	0.252
	T3	1.19 (0.72–1.95)	0.496	1.20 (0.73–1.98)	0.479	1.48 (0.85–2.59)	0.165
NAR	Continuous	1.50 (1.10–2.04)	0.011	1.65 (1.19–2.28)	0.002	1.32 (0.94–1.85)	0.112
Tertiles	T1	1.00		1.00		1.00	
	T2	1.20 (0.72–2.01)	0.485	1.23 (0.73–2.06)	0.439	0.87 (0.50–1.52)	0.630
	T3	1.69 (1.03–2.75)	0.037	1.93 (1.17–3.18)	0.010	1.16 (0.68–1.96)	0.591
LAR	Continuous	0.68 (0.51–0.91)	0.010	0.71 (0.52–0.96)	0.026	0.91 (0.69–1.22)	0.536
Tertiles	T1	1.00		1.00		1.00	
	T2	0.65 (0.40–1.04)	0.071	0.65 (0.40–1.05)	0.079	0.82 (0.50–1.36)	0.451
	T3	0.55 (0.34–0.90)	0.017	0.60 (0.36–0.99)	0.045	0.72 (0.43–1.21)	0.214
MAR	Continuous	1.18 (0.88–1.58)	0.258	1.20 (0.89–1.60)	0.227	1.17 (0.89–1.52)	0.258
Tertiles	T1	1.00		1.00		1.00	
	T2	0.93 (0.55–1.58)	0.783	0.90 (0.53–1.52)	0.687	0.94 (0.55–1.61)	0.827
	T3	1.68 (1.04–2.71)	0.033	1.71 (1.06–2.76)	0.028	1.65 (1.01–2.69)	0.046
RAR	Continuous	4.55 (2.12–9.77)	<0.001	5.84 (2.65–12.89)	<0.001	3.66 (1.42–9.40)	0.007
Tertiles	T1	1.00		1.00		1.00	
	T2	1.88 (1.10–3.22)	0.021	1.86 (1.09–3.19)	0.024	1.56 (0.88–2.74)	0.125
	T3	2.23 (1.31–3.77)	0.003	2.52 (1.48–4.30)	<0.001	1.87 (1.04–3.36)	0.037

After excluding patients who died within 7 days, a total of 452 patients remained, including 67 non-survivors and 385 survivors. After excluding patients with malignant tumors, a total of 457 patients remained, including 97 non-survivors and 360 survivors.

Model 1: no covariates were adjusted.

Model 2: age, gender, and weight were adjusted.

Model 3: MIMIC cohort: age, weight, SBP, DBP, anion gap, urea nitrogen, creatinine, potassium, SOFA, SAPS II, OASIS, sepsis, drug antibiotic used, and valve surgery were adjusted. The validation cohort: age, gender, weight, SOFA score, and valve surgery were adjusted.

## Discussion

In the present study, we investigated the prognostic value of five albumin-related inflammatory biomarkers (NAR, LAR, MAR, PAR, and RAR) in patients with rheumatic heart disease using both the MIMIC-IV database and an independent external validation cohort from China. Our findings demonstrated that RAR exhibited the most robust and consistent association with 30-day mortality across both cohorts. Specifically, RAR was significantly associated with increased mortality risk in the fully adjusted Cox model, showed a significant positive linear dose–response relationship, achieved the highest discriminative ability on time-dependent ROC analysis, and provided significant incremental predictive value beyond established severity scores in both cohorts.

Albumin-related inflammatory biomarkers have demonstrated prognostic value across diverse clinical domains. In respiratory diseases, RAR can predict the mortality rate of critically ill lung cancer patients (26). In oncology, the LAR independently predicted survival in nasopharyngeal carcinoma, while the PAR predicted postoperative outcomes in hepatocellular carcinoma (27, 28). In cardiovascular medicine, NAR has been proven to be a reliable biomarker for predicting long-term outcomes of CHD (29). Notably, in critical care, the PAR independently predicted acute kidney injury development and in-hospital mortality following cardiac surgery (30). Our study extends this established framework to RHD, where RAR emerged as the most robust predictor of short-term mortality.

The association between RAR and 30-day mortality in RHD patients may stem from the following reasons. First, the RDW is a simple and cost-effective parameter that reflects the degree of heterogeneity in red blood cell volume (31). In acute and chronic inflammatory conditions, pro-inflammatory cytokines suppress erythrocyte maturation, leading to the premature release of immature red blood cells into the circulation and consequent anisocytosis (32). In RHD patients, persistent autoimmune inflammation following Group A Streptococcus infection creates a chronic inflammatory milieu that likely drives RDW elevation. Second, albumin functions as a potent scavenger of pro-inflammatory mediators, including endotoxins, free fatty acids, and pro-inflammatory cytokines, thereby attenuating systemic inflammation, hypoalbuminemia signifies impaired anti-inflammatory and antioxidant defenses (33). It also serves as the primary antioxidant in plasma, neutralizing reactive oxygen species and protecting vascular endothelial integrity (34). Reduced albumin levels in RHD patients may therefore reflect both nutritional depletion and diminished capacity to counteract inflammation-driven tissue damage. RAR integrates these two critical indicators; elevated RAR levels simultaneously reflect both systemic inflammatory status and nutritional deficiencies, which are hallmark features of advanced RHD patients (35, 36).

External validation of RAR’s prognostic utility across various healthcare systems demonstrates that this biomarker reflects underlying pathological biological processes. Deriving from routinely available and cost-effective laboratory parameters, it further enhances its clinical application value in resource-limited regions, where the burden of rheumatic heart disease remains highest.

The following limitations of this study should be acknowledged. First, the retrospective observational design precludes the establishment of causal relationships between albumin-related inflammatory ratios and mortality, and residual confounding from unmeasured variables cannot be entirely excluded despite multivariable adjustment. Second, this study is limited by its reliance on a single baseline measurement to assess albumin-related inflammatory markers. Future prospective studies should evaluate the prognostic value of these biomarkers’ dynamic changes. Third, the primary endpoint was limited to 30-day mortality; longer-term outcomes were not evaluated. Fourth, the pivotal role of C-reactive protein-mediated acute-phase inflammatory responses and globulin-associated immunomodulatory effects in the course of RHD underscores the significance of both the C-reactive protein-to-albumin ratio (CAR) and the albumin-to-globulin ratio (AGR) as biologically meaningful biomarkers in the field of RHD. However, due to excessively high rates of CRP and globulin deficiency in both cohorts, these parameters were excluded from the analysis. Future studies incorporating comprehensive CRP and globulin testing data should directly compare these two biomarkers to identify the optimal inflammation-nutrition index for RHD risk stratification. Fifth, although the baseline characteristics of the two cohorts are comparable, unmeasured background factors (socialeconomic status, insurance status, severity grading of rheumatic heart disease, prophylactic medication use, etc.) may affect the generalizability of the study finding. Sixth, we acknowledge that deaths from non-cardiac causes may act as competing risks in this critically ill cohort. Unfortunately, reliable cause-of-death data were unavailable, preventing formal competing risk analyses. Seventh, the sensitivity analyses excluding early deaths and malignant tumors yielded restricted sub-cohorts with altered event compositions, which may affect limit direct comparability with the primary cohort; thus, these findings should be interpreted with caution. Future prospective multicenter studies with serial biomarker measurements and extended follow-up are warranted to confirm our findings.

## Conclusion

In this dual-cohort study of RHD patients, RAR emerged as the most robust and consistently validated predictor of 30-day mortality. Derived from routine laboratory parameters, RAR offers a simple, cost-effective tool for early risk stratification. Prospective studies are required to validate our findings.

## Data Availability

The raw data supporting the conclusions of this article will be made available by the authors, without undue reservation.

## Glossary

CHD: Coronary heart disease
CI: Confidence interval
DBP: Diastolic blood pressure
DM: Diabetes mellitus
HELLP: Hemolysis, elevated liver enzymes, and low platelet count
HR: Hazard ratio
ICD-9: International Classification of Diseases, 9th Revision
ICD-10: International Classification of Diseases, 10th Revision
IDI: Integrated discrimination improvement
IQR: Interquartile range
LAR: Lymphocyte-to-albumin ratio
MAR: Monocyte-to-albumin ratio
MIMIC-IV: Medical Information Mart for Intensive Care-IV
NAR: Neutrophil-to-albumin ratio
OASIS: Oxford Acute Severity of Illness Score
PAR: Platelet-to-albumin ratio
RAR: Red blood cell distribution width-to-albumin ratio
RCS: Restricted cubic spline
RDW: Red blood cell distribution width
RHD: Rheumatic heart disease
ROC: Receiver operating characteristic
SAPS II: Simplified Acute Physiology Score II
SBP: Systolic blood pressure
SD: Standard deviation
SOFA: Sequential Organ Failure Assessment

## References

[1] Zou Y Wei S Ren Q Zhou T Lu S Li R . Global burden of rheumatic heart disease (1990-2021), attributable risk factors, and future projections. JACC Basic Transl Sci. (2026) 11:101511. doi: 10.1016/j.jacbts.2026.101511, 41780208 PMC12969019

[2] Lupieri A Jha PK Nizet V Dutra WO Nunes MCP Levine RA . Rheumatic heart valve disease: navigating the challenges of an overlooked autoimmune disorder. Front Cardiovasc Med. (2025) 12:1537104. doi: 10.3389/fcvm.2025.1537104, 40182432 PMC11966398

[3] Li C Lei S Liu L Yuan Y Tian J. The burden of cardiovascular disease in children in Asian countries (1990-2021): systematic analysis and projection of the burden of disease. Am J Prev Cardiol. (2025) 21:100956. doi: 10.1016/j.ajpc.2025.100956, 40135151 PMC11932875

[4] Mbanze I Spracklen TF Jessen N Damasceno A Sliwa K. Heart failure in low-income and middle-income countries. Heart. (2025) 111:341–51. doi: 10.1136/heartjnl-2024-324176, 40010938

[5] Ruan R Liu X Zhang Y Tang M He B Zhang QW . Global, regional, and National Advances toward the Management of Rheumatic Heart Disease Based on the global burden of disease study 2019. J Am Heart Assoc. (2023) 12:e028921. doi: 10.1161/JAHA.122.028921, 37366108 PMC10356074

[6] Watkins DA Johnson CO Colquhoun SM Karthikeyan G Beaton A Bukhman G . Global, regional, and National Burden of rheumatic heart disease, 1990-2015. N Engl J Med. (2017) 377:713–22. doi: 10.1056/NEJMoa1603693, 28834488

[7] Hauge SW Estensen ME Persson RM Yadeta D Oljira CF Tessema A . Mid-term outcomes after open-heart surgery for severe chronic rheumatic heart disease in a low-income country: an observational study with historical controls. Front Cardiovasc Med. (2026) 13:1763889. doi: 10.3389/fcvm.2026.1763889, 41890879 PMC13013438

[8] Nasir M Markos S. Survival and mortality predictors among adults with rheumatic heart disease in Ethiopia: a 10-year retrospective cohort study. BMC Cardiovasc Disord. (2026) 26:383. doi: 10.1186/s12872-026-05751-7, 41877058 PMC13137564

[9] Molla FA Ayalew DG Astatk HA Teshager AW Legesse GL Agonafir DB . Prevalence and determinants of pulmonary hypertension in rheumatic heart disease patients at university of Gondar comprehensive specialized hospital: a retrospective study from 2018 to 2023. BMC Cardiovasc Disord. (2025) 25:193. doi: 10.1186/s12872-025-04648-1, 40102731 PMC11916979

[10] Olsen J Chimalizeni Y Carapetis J Chiume M Gunter SM Hosseinipour M . Distance from tertiary care, pericardial effusion, and nutritional status predict all-cause mortality among Malawian children with rheumatic heart disease. medRxiv. (2026). doi: 10.64898/2026.02.28.26346960

[11] Bernardi M Angeli P Claria J Moreau R Gines P Jalan R . Albumin in decompensated cirrhosis: new concepts and perspectives. Gut. (2020) 69:1127–38. doi: 10.1136/gutjnl-2019-318843, 32102926 PMC7282556

[12] Wu L Han D Xue Y He S Ma Z Su S . Association between the C-reactive protein-albumin-lymphocyte index and metabolic syndrome: evidence from the 2003-2010 national health and nutrition examination survey. Diabetol Metab Syndr. (2025) 17:39. doi: 10.1186/s13098-025-01609-8, 39891279 PMC11783767

[13] Kindan A Kurt DS Nalbantcilar DR Ulusoy CO Yucel KY. Evaluation of albumin-based inflammatory markers as diagnostic tools in HELLP syndrome. Sci Rep. (2025) 15:22125. doi: 10.1038/s41598-025-01797-3, 40593842 PMC12218002

[14] Wang MD Duan FF Hua X Cao L Xia W Chen JY. A novel albumin-related nutrition biomarker predicts breast Cancer prognosis in Neoadjuvant chemotherapy: a two-center cohort study. Nutrients. (2023) 15:4292. doi: 10.3390/nu15194292, 37836576 PMC10574703

[15] Zhang CL Gao MQ Jiang XC Pan X Zhang XY Li Y . Research progress and value of albumin-related inflammatory markers in the prognosis of non-small cell lung cancer: a review of clinical evidence. Ann Med. (2023) 55:1294–307. doi: 10.1080/07853890.2023.2192047, 37036321 PMC10088931

[16] Xiao L Li F Sheng Y Hou X Liao X Zhou P . Predictive value analysis of albumin-related inflammatory markers for short-term outcomes in patients with in-hospital cardiac arrest. Expert Rev Clin Immunol. (2025) 21:249–57. doi: 10.1080/1744666X.2024.2399700, 39223971

[17] Ji H Wang Y Cao X Liu Y Xu M Zhao X . Neutrophil percentage to albumin ratio predicts cardiovascular and all-cause mortality in diabetes and pre diabetes patients. Sci Rep. (2025) 15:10075. doi: 10.1038/s41598-025-93558-5, 40128326 PMC11933700

[18] Arba IF Multazam C Widiarti W Siahaan PP Oktaviono YH Setyobudi D . A systematic review and dose-response meta-analysis of red blood cell distribution width to albumin ratio as mortality predictor in cardiovascular disease. Sci Rep. (2025) 15:32053. doi: 10.1038/s41598-024-81876-z, 40890156 PMC12402460

[19] Li C Xu X Ma M Ma H. Platelet-albumin ratio predicts 28-day mortality risk in ICU heart failure patients: a retrospective cohort study. Medicine (Baltimore). (2026) 105:e46885. doi: 10.1097/MD.0000000000046885, 41496129 PMC12778142

[20] Hao M Jiang S Tang J Li X Wang S Li Y . Ratio of red blood cell distribution width to albumin level and risk of mortality. JAMA Netw Open. (2024) 7:e2413213. doi: 10.1001/jamanetworkopen.2024.13213, 38805227 PMC11134218

[21] Johnson AEW Bulgarelli L Shen L Gayles A Shammout A Horng S . MIMIC-IV, a freely accessible electronic health record dataset. Sci Data. (2023) 10:1. doi: 10.1038/s41597-022-01899-x, 36596836 PMC9810617

[22] Bland JM Altman DG. Transformations, means, and confidence intervals. BMJ. (1996) 312:1079. doi: 10.1136/bmj.312.7038.1079, 8616417 PMC2350916

[23] Mutagaywa RK Wind AM Kamuhabwa A Cramer MJ Chillo P Chamuleau S. Rheumatic heart disease anno 2020: impacts of gender and migration on epidemiology and management. Eur J Clin Investig. (2020) 50:e13374. doi: 10.1111/eci.13374, 32789848 PMC7757241

[24] Shariq T Rahman AS Jamal Q Siddiqui M Khan U Taufiq F. The SOFA score as a mortality predictor in ICU patients: insights from a tertiary care hospital in Pakistan. BMC Res Notes. (2025) 19:12. doi: 10.1186/s13104-025-07596-3, 41353387 PMC12784596

[25] Russell EA Tran L Baker RA Bennetts JS Brown A Reid CM . A review of outcome following valve surgery for rheumatic heart disease in Australia. BMC Cardiovasc Disord. (2015) 15:103. doi: 10.1186/s12872-015-0094-1, 26399240 PMC4580994

[26] Zhang L Liu T Wang G Liu B Shen H Mei Y . The red cell distribution width to albumin ratio as a novel predictor of 180-day mortality in lung cancer patients. Sci Rep. (2026) 16:4773. doi: 10.1038/s41598-026-35005-7, 41495393 PMC12873277

[27] Peng RR Liang ZG Chen KH Li L Qu S Zhu XD. Nomogram based on lactate dehydrogenase-to-albumin ratio (LAR) and platelet-to-lymphocyte ratio (PLR) for predicting survival in nasopharyngeal carcinoma. J Inflamm Res. (2021) 14:4019–33. doi: 10.2147/JIR.S322475, 34447260 PMC8385134

[28] Li C Peng W Zhang XY Wen TF Chen LP. The preoperative platelet to albumin ratio predicts the prognosis of hepatocellular carcinoma patients without portal hypertension after liver resection. Medicine. (2019) 98:e17920. doi: 10.1097/MD.0000000000017920, 31702672 PMC6855578

[29] Wu H Feng K Hua Y Lin Z Ding N Xie Y . Neutrophil-to-albumin ratio predicts long-term prognosis in coronary heart disease: a prospective cohort study of 2,990 patients. Front Nutr. (2025) 12:1674969. doi: 10.3389/fnut.2025.1674969, 41479660 PMC12754729

[30] He Z Wang H Wang S Li L. Predictive value of platelet-to-albumin ratio (PAR) for the cardiac-associated acute kidney injury and prognosis of patients in the intensive care unit. Int J Gen Med. (2022) 15:8315–26. doi: 10.2147/IJGM.S389846, 36451801 PMC9704005

[31] Garcia-Escobar A Lazaro-Garcia R Goicolea-Ruigomez J Gonzalez-Casal D Fontenla-Cerezuela A Soto N . Red blood cell distribution width is a biomarker of red cell dysfunction associated with high systemic inflammation and a prognostic marker in heart failure and cardiovascular disease: a potential predictor of atrial fibrillation recurrence. High Blood Press Cardiovasc Prev. (2024) 31:437–49. doi: 10.1007/s40292-024-00662-0, 39031283

[32] Song Y Li W Xiao J Cai G. Preoperative red cell width distribution to albumin ratio predicts early radial artery occlusion following coronary intervention via conventional transradial access. Sci Rep. (2025) 15:44093. doi: 10.1038/s41598-025-27940-8, 41413438 PMC12715189

[33] Chen ZB Wang ZW Ding CY Yan JH Gao Y Zhang Y . Can albumin administration relieve lung injury in trauma/hemorrhagic shock? World J Gastroenterol. (2006) 12:6884–8. doi: 10.3748/wjg.v12.i42.6884, 17106942 PMC4087448

[34] Alderson P Bunn F Lefebvre C Li WP Li L Roberts I . Human albumin solution for resuscitation and volume expansion in critically ill patients. Cochrane Database Syst Rev. (2002):CD001208. doi: 10.1002/14651858.CD001208 11869596

[35] Desta TT Gezachew A Eshetu K. Descriptive analysis of rheumatic heart disease related complications in pediatric patients at tertiary hospital, Addis Ababa, Ethiopia. Pediatr Health Med Ther. (2023) 14:45–57. doi: 10.2147/PHMT.S396854 PMC9930578 36817760

[36] Thorup L Hamann SA Tripathee A Koirala B Gyawali B Neupane D . Evaluating vitamin D levels in rheumatic heart disease patients and matched controls: a case-control study from Nepal. PLoS One. (2020) 15:e0237924. doi: 10.1371/journal.pone.0237924, 32822412 PMC7444549

