# Supplementary material for: Prognostic value of albumin-related inflammatory ratios for 30-day mortality in rheumatic heart disease: a dual-cohort study with external validation
Source: Front Nutr. 2026 Aug 13;13:1916028. doi: 10.3389/fnut.2026.1916028 (PMC13518121; doi:10.3389/fnut.2026.1916028)
Supplement: Supplementary file 1 [file Supplementary_file_1.DOCX]

**Supplementary Materials**

**Table S1. Baseline characteristics of RHD patients grouped according to different cohort;**

**Table S2. Missing data proportions for all covariates in the MIMIC-IV.**

| **Table S1. Baseline characteristics of RHD patients grouped according to different cohort** | | | | |
| --- | --- | --- | --- | --- |
| Variables | Total  (n = 764) | MIMIC cohort  (n = 496) | Validation cohort  (n = 268) | P |
| Age, years | 75.53 ± 14.15 | 75.97 ± 13.93 | 74.73 ± 14.55 | 0.248 |
| Gender, % |  |  |  | 0.321 |
| Female | 412 (53.93) | 274 (55.24) | 138 (51.49) |  |
| Male | 352 (46.07) | 222 (44.76) | 130 (48.51) |  |
| Weight, Kg | 75.77 ± 19.75 | 75.39 ± 19.82 | 76.46 ± 19.64 | 0.475 |
| Sodium, mmol/L | 136.13 ± 5.60 | 136.05 ± 5.45 | 136.29 ± 5.88 | 0.568 |
| Potassium, mmol/L | 3.99 ± 0.60 | 3.98 ± 0.61 | 4.00 ± 0.59 | 0.710 |
| Calcium, (mg/dL) | 8.24 ± 0.86 | 8.24 ± 0.83 | 8.25 ± 0.91 | 0.964 |
| Anion gap, mmol/L | 13.74 ± 3.71 | 13.74 ± 3.77 | 13.74 ± 3.61 | 0.981 |
| Urea nitrogen, mg/dl | 34.76 ± 24.33 | 34.96 ± 24.83 | 34.37 ± 23.42 | 0.750 |
| Glucose, mg/dl | 116.09 ± 41.22 | 115.28 ± 40.17 | 117.57 ± 43.12 | 0.465 |
| Creatinine, mg/dl | 1.73 ± 1.62 | 1.73 ± 1.62 | 1.71 ± 1.62 | 0.870 |
| Albumin, g/dl | 3.24 ± 0.60 | 3.25 ± 0.59 | 3.22 ± 0.61 | 0.474 |
| Platelet, K/µl | 173.14 ± 90.53 | 175.63 ± 93.45 | 168.52 ± 84.83 | 0.300 |
| RDW, % | 16.13 ± 2.69 | 16.13 ± 2.65 | 16.12 ± 2.77 | 0.969 |
| Neutrophil, K/uL | 10.03 ± 6.45 | 10.13 ± 6.48 | 9.85 ± 6.39 | 0.563 |
| Monocyte, K/uL | 0.69 ± 0.53 | 0.71 ± 0.59 | 0.65 ± 0.40 | 0.148 |
| Lymphocyte, K/uL | 1.26 ± 1.08 | 1.23 ± 1.03 | 1.31 ± 1.15 | 0.332 |
| NAR | 0.97 ± 0.67 | 0.98 ± 0.66 | 0.96 ± 0.68 | 0.644 |
| LAR | -1.16 ± 0.68 | -1.18 ± 0.68 | -1.11 ± 0.69 | 0.178 |
| MAR | -1.76 ± 0.73 | -1.74 ± 0.73 | -1.80 ± 0.74 | 0.257 |
| PAR | 3.86 ± 0.58 | 3.87 ± 0.59 | 3.85 ± 0.57 | 0.671 |
| RAR | 1.61 ± 0.27 | 1.61 ± 0.27 | 1.62 ± 0.27 | 0.624 |
| Respiratory rate, minute | 13.00 ± 3.80 | 12.92 ± 3.77 | 13.13 ± 3.87 | 0.460 |
| SBP, mmHg | 86.82 ± 16.39 | 86.70 ± 16.49 | 87.03 ± 16.21 | 0.793 |
| DBP, mmHg | 43.69 ± 11.15 | 43.66 ± 11.08 | 43.75 ± 11.30 | 0.915 |
| Heart rate, minute | 70.17 ± 17.12 | 70.27 ± 16.84 | 69.98 ± 17.65 | 0.826 |
| SOFA | 5.94 ± 3.49 | 5.90 ± 3.51 | 6.01 ± 3.45 | 0.684 |
| SAPS II | 43.05 ± 13.30 | 43.07 ± 13.13 | 43.02 ± 13.63 | 0.961 |
| OASIS | 33.92 ± 8.46 | 34.00 ± 8.48 | 33.77 ± 8.44 | 0.723 |
| Hypertension, % |  |  |  | 0.896 |
| No | 568 (74.35) | 368 (74.19) | 200 (74.63) |  |
| Yes | 196 (25.65) | 128 (25.81) | 68 (25.37) |  |
| Diabetes, % |  |  |  | 0.496 |
| No | 494 (64.66) | 325 (65.52) | 169 (63.06) |  |
| Yes | 270 (35.34) | 171 (34.48) | 99 (36.94) |  |
| Congestive heart failure, % |  |  |  | 0.896 |
| No | 169 (22.12) | 109 (21.98) | 60 (22.39) |  |
| Yes | 595 (77.88) | 387 (78.02) | 208 (77.61) |  |
| Drug antibiotic used, % |  |  |  | 0.986 |
| No | 197 (25.79) | 128 (25.81) | 69 (25.75) |  |
| Yes | 567 (74.21) | 368 (74.19) | 199 (74.25) |  |
| Valve surgery, % |  |  |  | 0.854 |
| No | 675 (88.35) | 439 (88.51) | 236 (88.06) |  |
| Yes | 89 (11.65) | 57 (11.49) | 32 (11.94) |  |
| 30-day mortality, % |  |  |  | 0.636 |
| No | 597 (78.14) | 385 (77.62) | 212 (79.10) |  |
| Yes | 167 (21.86) | 111 (22.38) | 56 (20.90) |  |
| RDW, red blood cell volume distribution width; PAR, platelet to albumin ratio; LAR, lymphocyte to albumin ratio; MAR, monocyte to albumin ratio; NAR, neutrophil to albumin ratio; RAR, red cell distribution width to albumin ratio; SBP, systolic blood pressure; DBP, diastolic blood pressure; SOFA, sequential organ failure assessment; SAPS II, simplified acute physiology; OASIS, oxford acute severity of illness score. | | | | |
| All values are expressed as a proportion (%) or mean ± standard deviation (SD). | | | | |

| Table S2. Missing data proportions for all covariates in the MIMIC-IV | |
| --- | --- |
| Variables | Missing data proportions (%) |
| Weight, Kg | 94.81 |
| Glucose, mg/dL | 99.20 |
| Calcium, mg/dL | 98.40 |
